# CLINIMETRIC PROPERTIES OF THE ABC-6, ABC-16, AND MODIFIED FALLS EFFICACY SCALE WHEN DELIVERED ELECTRONICALLY

**DOI:** 10.1093/geroni/igae098.3363

**Published:** 2024-12-31

**Authors:** Jennifer Blackwood, Matthew Carpenter

**Affiliations:** University of Michigan, Ann Arbor, Michigan, United States; University of Michigan Flint, Flint, Michigan, United States

## Abstract

The Activities Specific Balance Confidence Scales (ABC, ABC-6) and the Modified Falls Efficacy Scale (M-FES) are outcome measures used to assess self-efficacy in maintaining balance in older adults. All three measures have established reliability and validity when delivered in a clinician-driven paper assessment. However, it is unknown what the clinimetric properties of these measures are when delivered electronically without verbal direction. Therefore, the purpose of this study is to establish the reliability, validity, standard error of measurement, and the minimal detectable change (95% confidence interval) of these measures when delivered electronically in community-dwelling older adults. 67 participants were recruited electronically through the University of Michigan’s Health Research platform. Older adult participants were English-speaking; aged 65+ years; had access to a computer or electronic device with reliable internet; and were diagnosed with various health conditions. A Qualtrics survey was distributed to participants that consisted of the ABC-16 and M-FES with written directions. Results from the 67 participants demonstrated excellent test-retest reliability (ICC2,1 = ≥0.94) and excellent internal consistency (α = ≥0.94) for all measures. The ABC-16 had a smaller SEM (3.85) and MDC95 (10.67) compared to the ABC-6 (SEM = 4.99, MDC95 = 13.83). For the MFES, the SEM was 0.27 and MDC95 was 0.75. All measures had significant Pearson correlations (p < 0.01) with the falls history indicating construct validity. These results indicate the ABC-16/6 and M-FES can be utilized in an electronic format when assessing older adults’ self-efficacy in balance.

